# Endovascular Fiducial Placement in Splenic Metastatic Disease as a Novel Option for Radiotherapy: A Case Report

**DOI:** 10.7759/cureus.77186

**Published:** 2025-01-09

**Authors:** Adriana C Timmers, Joost Nuyttens, Mark A de Wolf

**Affiliations:** 1 Radiology and Nuclear Medicine, Erasmus Medical Center Cancer Institute, Rotterdam, NLD; 2 Radiation Oncology, Erasmus Medical Center Cancer Institute, Rotterdam, NLD

**Keywords:** colon carcinoma, fiducial marker placement, radiological intervention, splenic metastasis, stereotactic radiotherapy

## Abstract

Splenic metastases are rare but difficult-to-treat entities, especially if they recur after initial surgery or ablation. They are particularly difficult to treat with radiation therapy due to their subdiaphragmatic location and the presence of respiratory excursions. This report describes a case in which an endovascular fiducial placement approach was used to mark the splenic metastasis prior to radiation therapy.

We present a 77-year-old male with an extensive history of colorectal metastatic disease. After hemicolectomy, the patient showed metastases - first in the spleen and then in the liver. These were treated with locoregional therapy, including radiofrequency ablation (RFA) for the spleen metastasis and RFA and microwave ablation (MWA) for the liver metastases. Contrast-enhanced computed tomography (CT) imaging, eight years after initial therapy, showed two new liver metastases and a recurrent metastasis in the spleen. Percutaneous ablation of the splenic metastasis was deemed too dangerous because of the subdiaphragmatic location of the spleen and the presence of respiratory motion, and the multidisciplinary tumor board, therefore, opted for radiotherapy. To guide radiotherapy, the interventional radiologist chose to place three microcoil fiducial markers (FMs) around the splenic lesion via an endovascular transradial approach. Radiotherapy was successful, and no recurrence of splenic metastasis has been observed during follow-up. In summary, transradial endovascular FM placement in splenic metastatic disease is technically possible from both an interventional radiological and a radiotherapy standpoint.

## Introduction

The treatment of splenic metastases can be difficult, particularly after initial surgery or ablation. Stereotactic radiotherapy can be an excellent treatment option; however, this type of radiotherapy does require prior placement of fiducial markers (FMs) to account for motion [1,2]. In this report, we describe the endovascular placement of these markers.

## Case presentation

A 77-year-old male with a history of cardiovascular disease, oropharyngeal cancer, and prostate cancer was referred to the Department of Interventional Radiology. Seven years prior, the patient was diagnosed with adenocarcinoma of the transverse colon and underwent a left-sided extensive hemicolectomy. After hemicolectomy, imaging demonstrated a solitary metastasis in the spleen, measuring a maximum of 16 mm. This metastasis was treated with radiofrequency ablation (RFA). Following this, hyperthermic intraperitoneal chemotherapy (HIPEC) was performed for peritoneal lesions. Contrast-enhanced computed tomography (CT), six years after the first presentation, showed two new liver metastases (segment 6, 16 mm, and segment 8, 14 mm) and a recurrent tumor in the spleen, measuring a maximum of 13 mm. Both hepatic metastases were treated with microwave ablation (MWA) (as, in our center, SBRT, or stereotactic body radiation therapy, is preserved as an eventual future treatment option). As discussed by the multidisciplinary tumor board, surgery or locoregional treatment of the spleen was not an option given the prior ablation in this region, and stereotactic radiotherapy was proposed to be the best treatment option (eventual systemic treatment was reserved as a last resort). In CyberKnife Robotic Radiotherapy (Accuray Inc., Sunnyvale, CA, USA), placement of FMs is necessary to optimize the dose intensity in the tumor and reduce the gradient of the dose in surrounding tissue. To mitigate the complications associated with percutaneous FM placement, an endovascular (transarterial) approach was chosen for FM delivery, comparable with fiducial placement in the lung.

**Figure 1:**
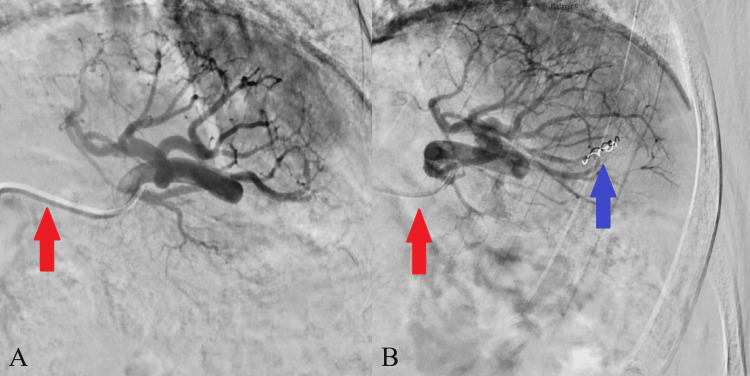
Selective angiography of the splenic artery Selective contrast-enhanced angiography of the splenic artery and side branches, pre-fiducial marker placement (A) and post-fiducial marker placement (B). The red arrows show the catheter in the splenic artery. The blue arrow shows the first fiducial placed in a splenic artery side branch.

Arterial access was established by an ultrasonography-guided puncture of the left radial artery at wrist level with a 21-gauge needle and the introduction of an 11 cm, 5-French sheath (Prelude; Merit Medical Systems, Inc., South Jordan, UT, USA). Afterward, a mixture of unfractionated heparin (5,000 units), verapamil (2.5 mg), and nitroglycerine (200 µg) was administered via the sheath. Catheterization of the descending aorta, celiac trunk, and splenic artery was performed with a 4-French, 125 cm braided catheter (Ultimate 1 Performa; Merit Medical Systems, Inc.) and a 180 cm Hydrophilic Coated Guidewire (Terumo Interventional Systems, Somerset, NJ, USA). Next, a microcatheter (Progreat; Terumo Interventional Systems) was used for selective catheterization and FM placement in the splenic artery side branches. Three FMs were placed at or near the margins of the metastasis (Figure 1). We chose to use platinum 2-3 mm spiral microcoils (Tornado Embolization Microcoil; Cook Medical, Bloomington, IN, USA), corresponding with the procedure for FM placement in the lung. The location of the microcatheter tip during catheterization and before placement of FMs was checked by using multi-angle fluoroscopy and cone-beam CT. Because of previous RFA, the region that housed the splenic metastasis was lacking side branches for fiducial placement; therefore, FMs were placed in splenic artery side branches adjacent to the lesion. Following this, the catheters and sheath were removed, and an airband (PreludeSYNC; Merit Medical Systems, Inc.) was placed as a vascular closure device for hemostasis at the puncture location and then deflated in the patient ward. After the FM placement, preventive analgesia (acetaminophen and oral opioids) was prescribed for possible ischemia-related pain.

Gross tumor volume (GTV) delineation was performed on the pre-interventional contrast-enhanced CT, taking into account the zone of prior ablation bordering the splenic metastasis. A 5-mm isotropic expansion from the volume of the spleen metastasis (GTV) was used for calculating the planned tumor volume (PTV). The three fiducial microcoil markers were located outside the GTV margins (within a 5-mm distance) and inside the PTV margins. A total dose of 45 Gy in five fractions was administered. In order to meet the constraints of the bowel (35 Gy <0.5 cc), the planning tumor volume was underdosed. The image-guided CyberKnife® system for robotic stereotactic radiotherapy and the Synchrony system (Accuray Inc.) for real-time respiratory tracking and compensation were used. The radiological response was noted using RECIST version 1.1.

**Figure 2:**
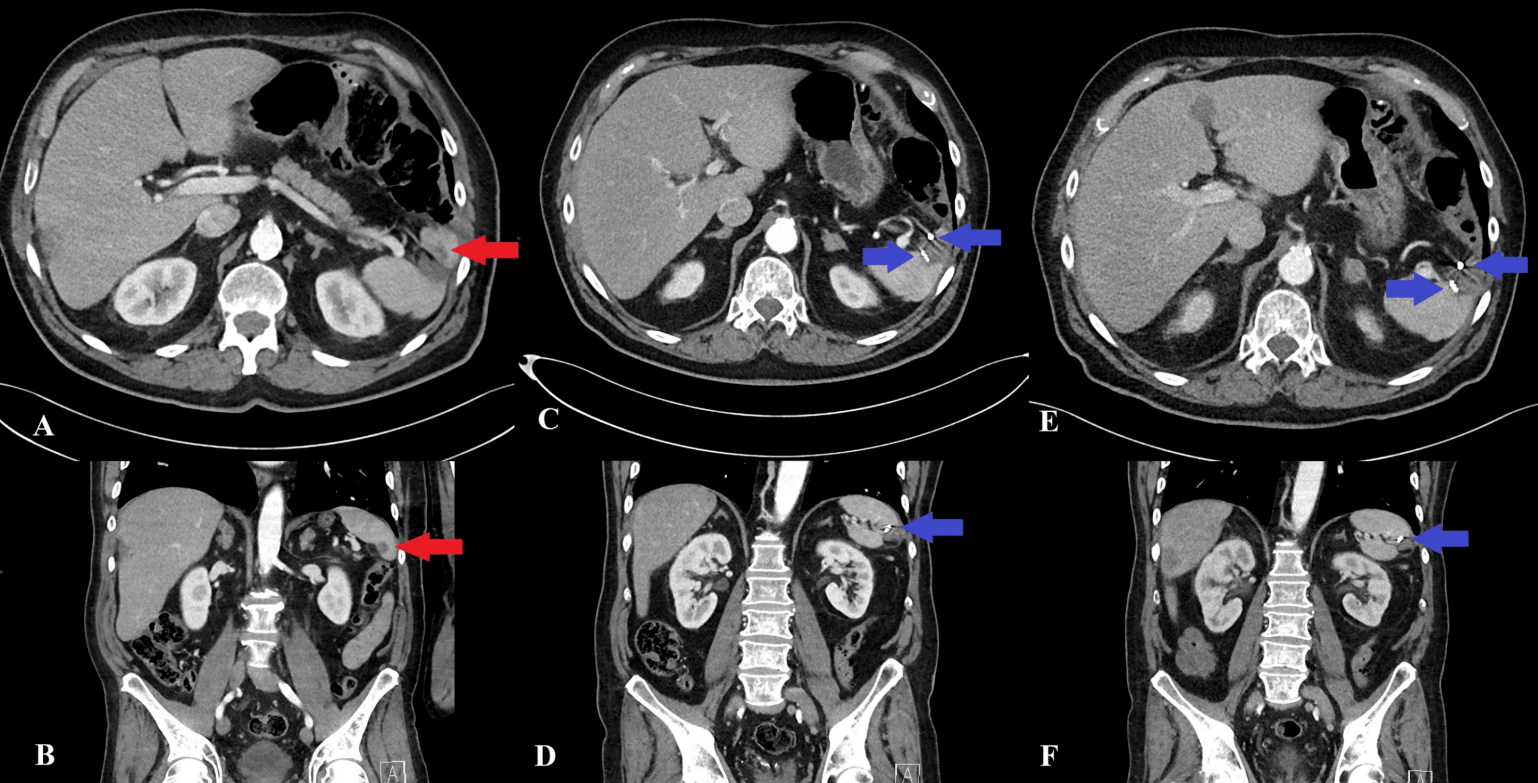
Computed tomography before and after fiducial placement Contrast-enhanced axial and coronal computed tomography scans of the abdomen pre-fiducial marker placement (A-B), post-fiducial marker placement (C-D), and the last follow-up control scan (E-F). The red arrows show the lesion, next to the ablation cavity. The blue arrows show the fiducial markers in the spleen.

Follow-up CT imaging was performed approximately every three months. During 19 months of follow-up, all CT scans showed successful treatment of the splenic metastasis, and no complications related to the FM placement or radiotherapy were noted (Figure 2). Unfortunately, multiple instances of liver metastases were noted on follow-up scans. On the first follow-up scan post-SBRT, four new liver metastases were diagnosed, and on subsequent CT scans, more new liver metastases were seen.

## Discussion

The present case report shows a rare case of splenic metastasis from colorectal cancer in a 77-year-old male. Endovascular FM placement, via a radial artery approach, for stereotactic radiotherapy guidance was used successfully.

Robotic stereotactic radiotherapy is an accurate treatment option for both primary tumors and metastasis, precisely delivering high single-dose or consecutive-dose radiation to a specific tumor location [1]. Dose targeting, to make sure that a maximum dose is delivered to the tumor while sparing the surrounding organs at risk, can be challenging. Respiratory movements, or involuntary movements of other organs, are factors that can reduce the target doses [2]. To overcome this problem, FMs should be placed within or near the primary tumor before radiation treatment. FMs can be inserted in a minimally invasive way into various targets via percutaneous or endoluminal access [3-7]. FM placement is widely described in patients with primary lung malignancy and in lung metastasis, where percutaneous placement has been largely abandoned in favor of endovascular placement due to a better safety profile.

FM placement is still a rare application in patients with splenic metastases. Trumm et al. [8] presented one case of CT fluoroscopy-guided transcutaneous FM placement in splenic metastasis prior to CyberKnife radiotherapy, in a large series of FM placements in various organs. Deshmukh et al. [9] described endoscopic ultrasound (EUS)-guided fiducial gold marker placement in metastatic colon cancer to the spleen, in a patient who refused a splenectomy. Partlow et al. [10] presented a case of transcutaneous FM placement in an omental metastasis near the spleen, using cone beam CT in a hybrid operating room, enabling laparoscopic resection. However, to the best of our knowledge, our case is the first in which an endovascular approach for FM placement has been chosen.

The limitations of endovascular FM placement are mostly related to vascular anatomy, since catheterization of the splenic side branches can be difficult or impossible in some patients after surgery, RFA, MWA, or prior radiotherapy. The advantage of endovascular placement is the decreased theoretical risk of splenic hemorrhage, due to avoiding perforation of the splenic capsule and traumatic puncture of splenic tissue, compared to percutaneous placement. Moreover, the risk of pneumothorax, which can be an issue when puncturing the cranial parts of the spleen, is nonexistent. However, specific arterial access complications, such as dissection of the radial artery, can be present. Nevertheless, radial access is associated with a risk reduction in vascular complications, bleeding, and mortality, compared to femoral access.

All types of FM delivery, including our case, showed excellent safety and technical results. Therefore, without any comparison studies, the technique of FM delivery should be based on patient and physician preference.

## Conclusions

Transradial endovascular FM placement in splenic metastatic disease is technically possible, both from an interventional radiological as well as a radiotherapy standpoint. This technique obviates the risks associated with percutaneous fiducial placement, mainly bleeding. In this case, treatment of the marked lesion by radiotherapy was technically and clinically successful, but, unfortunately, metastases in the liver recurred in the patient.
